# Cervical kyphosis surgery using a cervical pedicle screw placed with a U-shaped wire that enables observation of the lateral edge of the cortical bone of the spinal canal: A case report and literature review

**DOI:** 10.1097/MD.0000000000036088

**Published:** 2023-11-17

**Authors:** Chikara Ushiku, Shoshi Akiyama, Tomoaki Kanai, Naomu Sawada, Mitsuru Saito

**Affiliations:** a Department of Orthopaedic Surgery, The Jikei University Kashiwa Hospital, Chiba, Japan; b Department of Orthopaedic Surgery, The Jikei University School of Medicine, Tokyo, Japan.

**Keywords:** case report, cervical pedicle screw, lateral end of the cortical bone of the spinal canal, U-shaped wire

## Abstract

**Rationale:**

A cervical pedicle screw (CPS) serves as an important anchor for cervical surgeries. Its placement requires the development of a highly safe and easy-to-handle method. Considering that the lateral end of the cortical bone of the spinal canal (LE point) is the most crucial for CPS placement, we devised a U-shaped wire capable of identifying LE points under direct vision and reliably confirming the site with C-arm lateral fluoroscopy.

**Patient concerns:**

A 65-year-old male, who had been aware of numbness in both hands, mild finger dexterity disorder, and gait disturbance for half a year, visited our hospital due to the progression of his symptoms in the previous 2 months.

**Diagnosis:**

The patient presented with mild muscle weakness and tendon hyperreflexia in the upper and lower extremities on both sides, and magnetic resonance imaging revealed moderate spinal canal stenosis at the C4/5 and 5/6 levels. Based on the local third cervical vertebra (C3)/4 angle of −10 degrees and the C2/7 angle of −15 degrees, the patient was diagnosed with cervical myelopathy with cervical kyphosis. He had a Japanese Orthopaedic Association score for cervical myelopathy of 10.

**Interventions:**

We placed CPSs at C3 using a U-shaped wire. After placing an anchor in the range of C3-T1, laminectomy from C4 to C7 was performed. Subsequently, corrective fixation was performed to reduce kyphosis, followed by bone grafting in the range of C3-T1 and complete posterior cervical decompression fixation.

**Outcomes:**

The CPSs were placed at C3 without deviation and intra- or postoperative complications. The surgery resulted in improvement in kyphosis with a C2/7 angle of −5 degrees and recovery in spinal cord disorder with a Japanese Orthopedic Association score for cervical myelopathy of 13.

**Lessons:**

A U-shaped wire, which can be prepared inexpensively and easily, is a useful tool, especially for inexperienced surgeons, for safe CPS placement by capture of LE points accurately.

## 1. Introduction

The pedicle screw (PS) method, which anchors a PS in the thick cortical bone at the inner margin of the pedicle, has considerably high pullout strength and stability against repeated loads compared to other methods of spinal fusion.^[1,2]^ Due to this superior mechanical property, the method is widely used for spinal fusion surgeries in cases of high instability and those requiring strong support, correction for such as deformity and dislocation. The PSs have a high correction maintenance effect.^[3]^ A cervical pedicle screw (CPS) serves as an important anchor in surgeries for cervical kyphosis and cervical spine/upper thoracic spine injuries. On the other hand, due to the proximity of the vertebral artery and nerve tissue at the cervical spine levels, deviation of CPS from the pedicle leads to a high risk of spinal cord and nerve root injuries, and causes life-threatening risks, such as injury of vertebral artery injury.^[4]^ Thus, CPS placement requires utmost precaution.

The method of capturing the anatomical structures around the pedicle in surgical field is an approach to ensure CPS placement with safety. Considering that the lateral end of the cortical bone of the spinal canal (LE point) is the most crucial for CPS placement, we devised a U-shaped wire for identifying LE points under direct vision and reliably confirming the site with C-arm lateral fluoroscopy. Here, we introduce a case of cervical kyphosis surgery in which CPSs were placed using this method.

## 2. Case report

**Figure 1.**
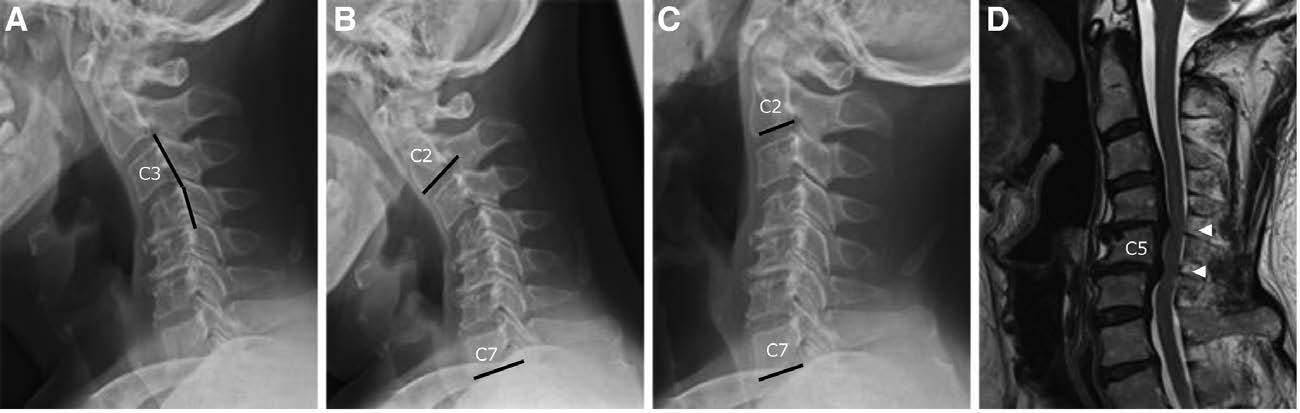
Preoperative images. (A) Neutral plain X-ray of the cervical spine. (B) Anteflexion position. (C) Retroflexion position. (D) MRI T2-weighted sagittal view.

The patient was a 65-year-old male who had been aware of numbness in both hands, mild finger dexterity disorder, and gait disturbance for half a year. He visited our hospital due to the progression of his symptoms in the previous 2 months. At the time of presentation at the hospital, the patient had muscle weakness [bilateral triceps manual muscle testing (MMT) 4 and little finger abductor muscle MMT 4], mild sensory disturbance (bilateral C6, 7, 8 regions), and tendon hyperreflexia in the upper and lower extremities on both sides (brachioradialis reflex, triceps reflex, patellar tendon reflex, and Achilles tendon reflex). The results of various tests were as follows: 10-seconds grip and release test (10-s test)^[5]^ showed a result of 8 cycles in the right hand and 6 cycles in the left hand, a Japanese Orthopedic Association score for cervical myelopathy (JOA) score^[6]^ of 10, and a Neck Disability Index (NDI)^[7]^ of 33. Neutral plain X-ray showed a local third cervical vertebra (C3)/4 angle of 10 degree kyphosis, a C2/7 angle of 15 degree kyphosis (Fig. 1A), and an anterior–posterior flexion motion range of 29 degrees (Fig. 1B and C). Anterior–posterior flexion dynamics had a reduced movement at C4/5/6, while there was mobility at C3/4. The C2-7 sagittal vertical axis was 29 mm, and the first thoracic vertebrae (T1) slope was 19 degrees (Fig. 1A). Magnetic resonance imaging revealed moderate spinal canal stenosis at the C4/5 and 5/6 levels (Fig. 1D).

Despite the presence of cervical kyphosis, there was no major vertebral body deformity or slippage. Thus, in addition to performing only posterior spinal decompression, C3-T1 posterior fixation was planned for the purpose of preventing alignment deterioration.

### 2.1. Preoperative preparation (Fig. 2)

**Figure 2.**
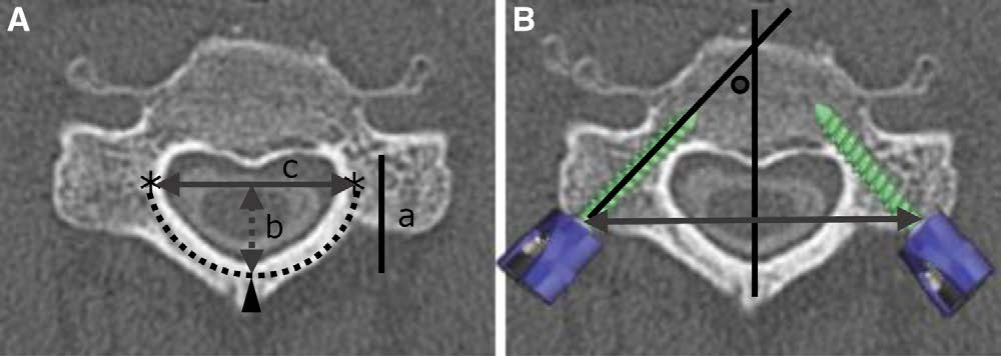
Preoperative measurements using CT images. (A and B) Transversal images at the level of C3 pedicle. (A) dotted line: Outer edge of the cortical bone, which is the wall forming the posterior part of the spinal canal. A, *: Left and right LE points. A, ▲: The most posterior point of the cortical bone of the spinal canal. Line a: Distance from the posterior wall of the vertebral body to the most posterior point of the cortical bone of the spinal canal. Double-arrow dotted line b: Distance between the line connecting the left and right LE points and the most posterior point of the cortical bone of the spinal canal. Double-arrow line c: Distance between the left and right LE points. B, ●: Insertion angle of a pedicle screw. B, double-arrow line: Width of the insertion point of pedicle screws.

On the computed tomography (CT) transversal images, at the level of the pedicle, where a PS was to be placed, we focused on the outer edge of the cortical bone, which was the wall forming the posterior part of the spinal canal (Fig. 2A, dotted line), and measured the distance (Fig. 2A, double-arrow) between the left and right LE points (Fig. 2A, *) and that (Fig. 2A, double-arrow dotted line) from the line connecting the left and right LE points to the most posterior point of the cortical bone of the spinal canal (Fig. 2A, ▲). The shape of the wire to be installed in the drilling of the cephalad side of the lamina was a U-shape traced along the shape of the posterior part of the spinal canal, connecting these 3, the LE points on the left and right sides and the most posterior point. For planning the placement site of a PS, the distance between the left and right insertion positions (Fig. 2B, double-arrow) and the insertion angle (Fig. 2B, ●) were measured.

### 2.2. Drilling in the lamina and development of LE points (Fig. 3)

The surgery was started under motor evoked potential monitoring. After exposing C3-7 from the vertebral arch to the entire lateral mass as well as T1 up to the base of the transverse process, PSs (ROBERT REID INC. S-shot) was placed at C7 and T1, and lateral mass screws were subsequently placed from C4 to C6.

**Figure 3.**
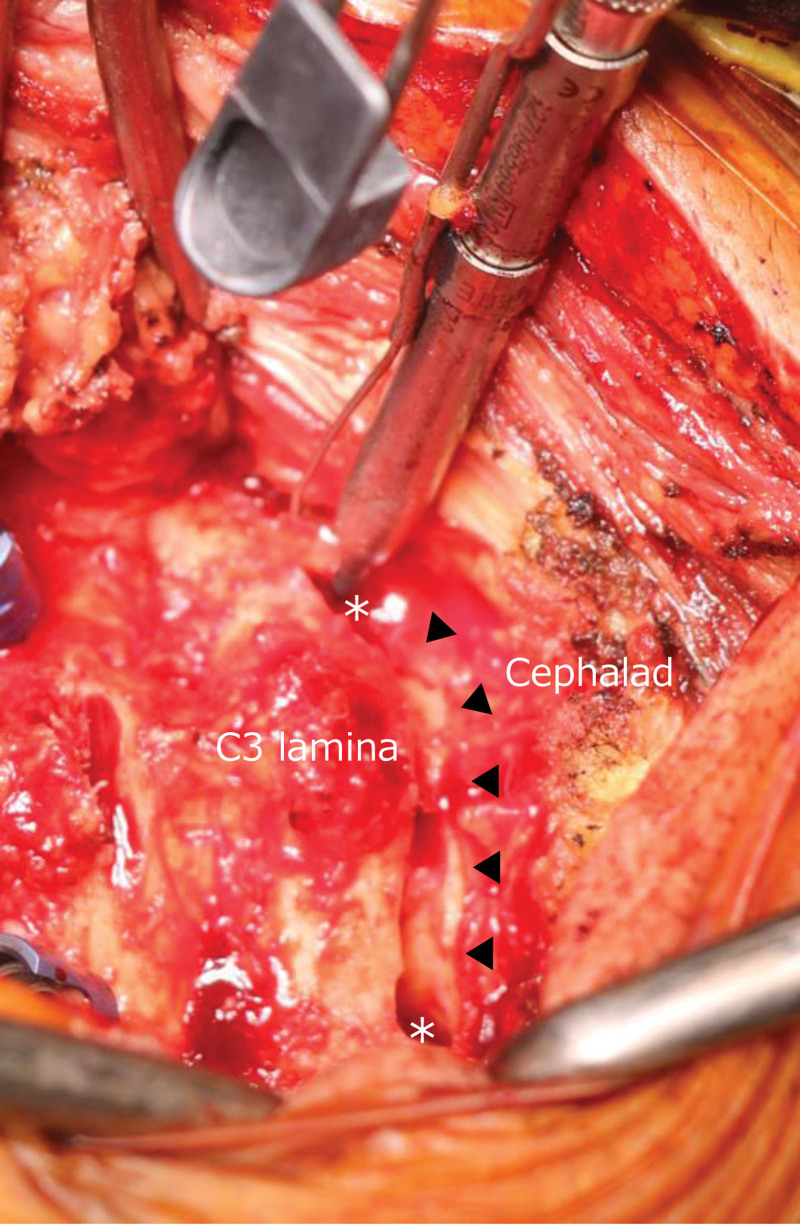
Drilling in the cephalad side of the lamina. ▲: Drilling site in the cephalad side of the C3 lamina. *: LE points.

Drilling in the cephalad side of the C3 lamina was started with a high-speed drill (4 mm diamond burr). The progression of drilling was as follows: from cortical bone on the dorsal side of the lamina to the cancellous bone along the shape of the posterior part of the spinal canal (Fig. 3) and the lateral side; the drilling was stopped at the LE points. Precaution was taken to ensure that the cortical bone on the ventral side of the lamina, which was the spinal canal wall, was not perforated with the drill.

### 2.3. Preparation of a U-shaped wire and its placement at the drilling site in the lamina (Fig. 4)

**Figure 4.**
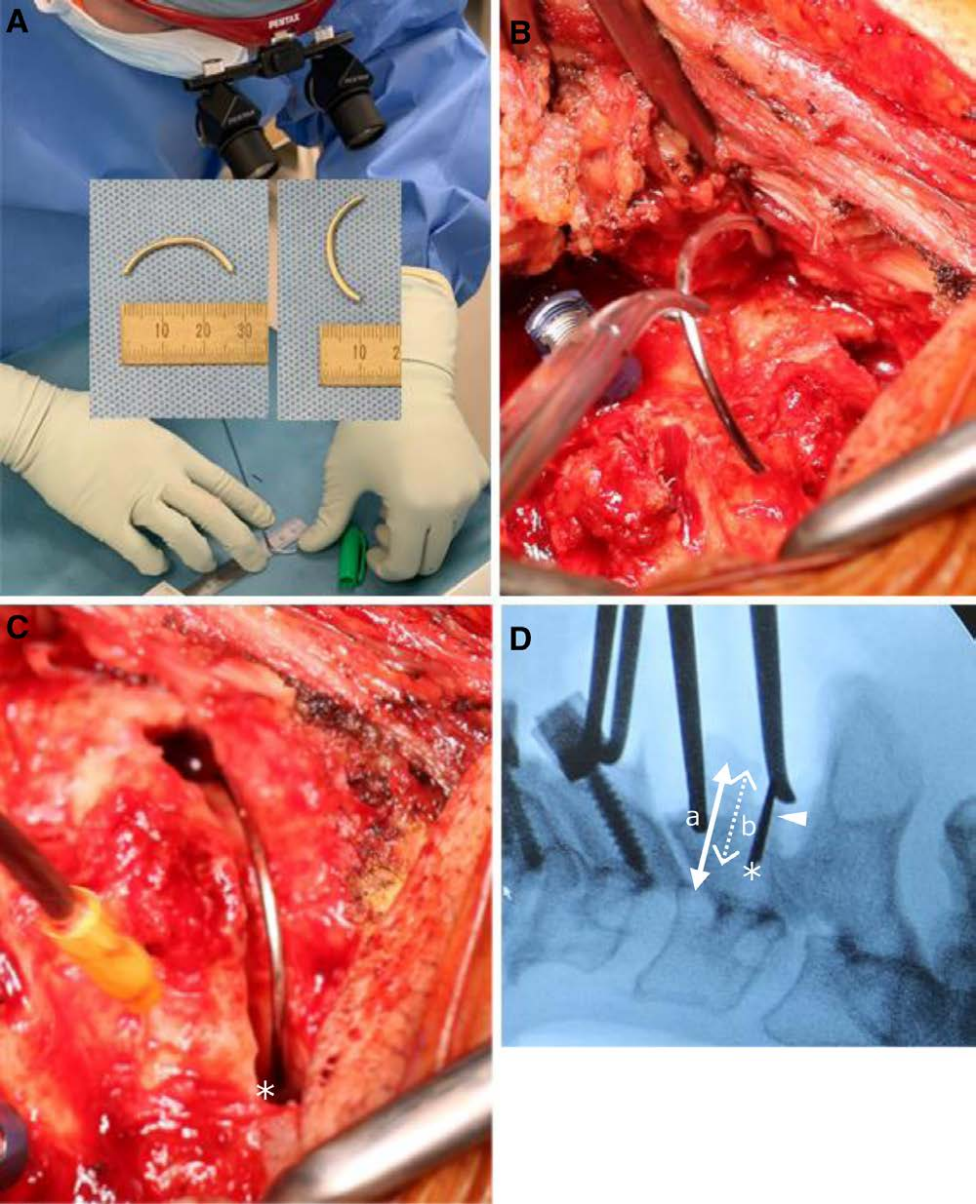
Preparation of a U-shaped wire and its placement in the lamina. A: Preparation process of a U-shaped wire and finished piece. B: Placement of a U-shaped wire at the drilling site in the lamina. C: Surgical field in which a U-shaped wire was placed at the drilling site in the lamina. D: Lateral fluoroscopic image of the placed wire. C, D *: LE points. D, △: U-shaped wire. D, double-arrow line a: Distance from the posterior wall of the vertebral body to the most posterior point of the cortical bone of the spinal canal. Double-arrow dotted line b: Distance between the line connecting the left and right LE points and the most posterior point of the cortical bone of the spinal canal.

A sterilized Kirschner steel wire (diameter: 1.4 mm) was shaped with a needle-nose plier and a wire cutter, along the U-shaped wire planned on the preoperative CT images (Fig. 4A). The U-shaped wire modeling the posterior part of the spinal canal was prepared for preoperative measurement. The important elements were the left and right LE points, and the most posterior point of the cortical bone of the spinal canal; we prioritized focusing on these 3 points. The wire was held with the surgical instrument mosquito (Fig. 4B), and was placed at the drilling site in the cephalad side of the C3 lamina (Fig. 4C). The wire was designed to model the anatomical shape of the posterior part of the spinal canal. If properly placed, the wire reliably confirms under direct vision that both ends of the wire are surely located at the LE points (Fig. 4C*). We determined the ratio between the distance from the most posterior point of the cortical bone of the spinal canal to the posterior wall of the vertebral body (Fig. 4D a) and the distance from the most posterior point of the cortical bone of the spinal canal to the line connecting the left and right LE points (Fig. 4D b); this ratio is designated as b/a. A comparison of this ratio with the ratio b/a presented in Fig. 2B reveals that the LE points present at the tips of the wire (Fig. 4D*) could also be confirmed under lateral fluoroscopy. Here, it is important to note that the image obtained with lateral fluoroscopy is a perfect lateral view for C3. Imprecise fitting of the wire in the posterior part of the spinal canal suggests that it has not reached the LE points, and drilling needs to be progressed deeper or craniocaudally to locate the point for precise fitting of the wire.

### 2.4. CPS placement with lateral fluoroscopy (Fig. 5)

**Figure 5.**
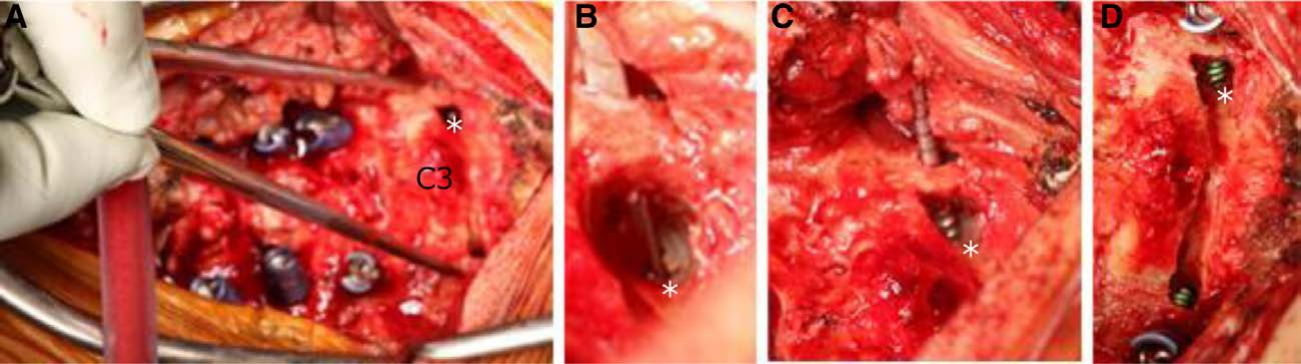
CPS placement. (A) Surgical field in which a caliper was aligned with the width of the insertion point. (B) Surgical field in which the probe was observed from the drilling site in the lamina. (C) Surgical field in which the tap was observed from the drilling site in the lamina. (D) Surgical field after the placement of a pedicle screw. A, B, C, D *: Lateral end point of the cortical bone of the spinal canal.

A caliper was aligned with the width of the preoperatively measured insertion point (Fig. 2B double-arrow black line, Fig. 5A), and a pilot hole was created in the C3 lateral mass with a high-speed drill. Advancement of the probe at an angle according to the preoperative plan helped in visual observation of the probe advancing immediately ahead of the LE points at the drilling site (Fig. 5B); the probe was progressed to the target depth by monitoring under lateral fluoroscopy. The direction of tap advancement followed the trajectory of probing; however, the tap advancing immediately ahead of the LE points could be visually observed from inside the drilling site (Fig. 5C). A PS was placed after confirming with a feeler that the created foramen was not perforated (Fig. 5D).

**Figure 6.**
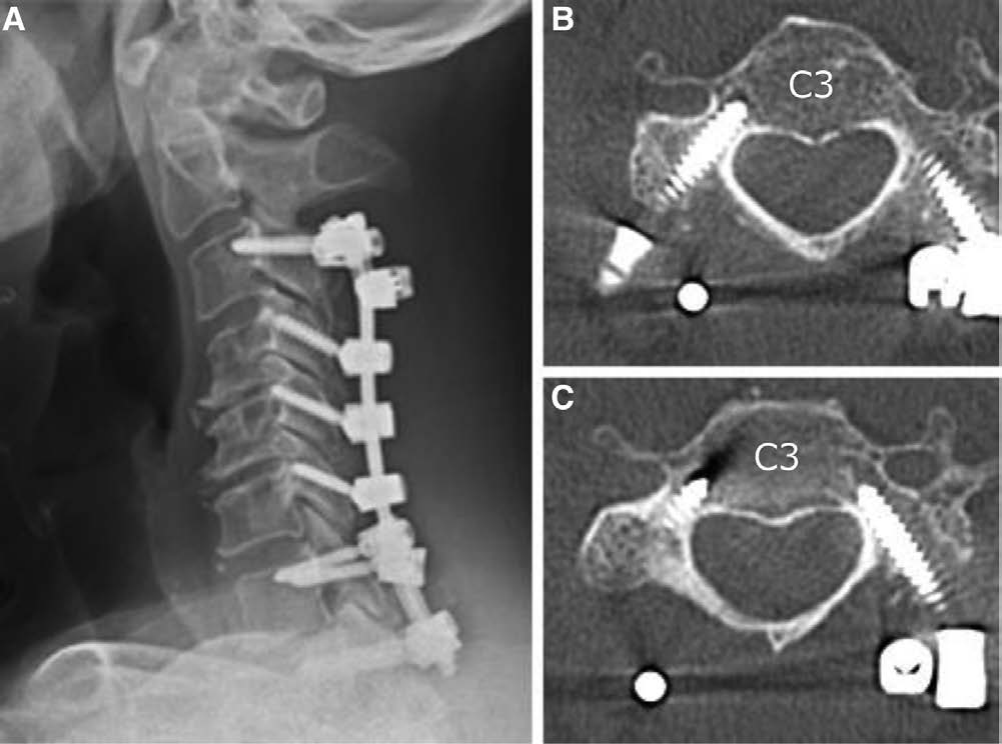
Images 1 month following surgery. (A) Neutral plain X-ray of the lateral side of the cervical spine. (B and C) Postoperative CT transversal images.

After placing PSs at C3, we performed laminectomy from C4 to C7. Subsequently, corrective fixation was performed to reduce kyphosis, and bone grafting was performed in the range of C3-T1. Deterioration of the motor evoked potential monitoring waveform did not occur during the surgery. The patient was able to resume his daily activities the day after surgery by wearing a cervical collar. At 1-month follow up after the surgery, his neck pain generally showed improvement with a C2/7 angle of 5-degree kyphosis, a C2/7 sagittal vertical axis of 10 mm, a T1 slope of 16 degrees (Fig. 6A), and an NDI of 11. The spinal cord disorder showed recovery with a 10-s test of 22 cycles in the right and 20 cycles in the left, and a JOA score of 13. The CPS was placed at C3 without deviation (Fig. 6B and C). The patient used a brace for up to 3 months postoperatively. At 6 months postoperatively, mild spinal cord impairment remained, with a JOA score of 15 owing to residual mild numbness and dysfunction in both hands. However, at a 10-s test of 25 cycles on both sides, his neck pain improved with an NDI score of 2 (very mild neck pain at the moment, and only do my usual work, but no more). No specific loss in the radiological parameters has occurred.

## 3. Discussion

The process of improving the safety of CPS placement can be divided into: the use of surgery-support devices and devising of surgical techniques that capture anatomical features in surgical field. The C-arm is presumed to be a surgery-support device for CPS placement that is most commonly used in general facilities. In terms of accuracy, CPS insertion under lateral fluoroscopy has been reported to have a deviation rate of 6.7% to 11.7%,^[8,9]^ and thus, its accuracy is not considered sufficient. Screw placement with lateral fluoroscopy is difficult in cases with severe bone deformity, such as rheumatoid arthritis, and cases of the cervicothoracic junction where visualization of the pedicle is difficult due to overlap with the shoulder line under lateral fluoroscopy. The fluoroscopic viewing placement method in the direction of the pedicle axis using the C-arm has been reported to have a CPS deviation rate of 8% to13.2%, indicating that its accuracy does not greatly differ from that of the lateral method.^[10]^ Surgery-support devices, other than C-arm, for improving insertion accuracy should include navigation^[11]^ and the use of three-dimensional patient-matched guides created from preoperative images,^[12]^ of superior quality for accurate screw placement and exposure reduction. However, these devices have limitations as the introduction of navigation is realistically difficult in terms of cost in many facilities, and the handling of patient-matched guides in emergency surgery is challenging.

Efforts have been made to improve the accuracy of PS placement, not by relying entirely on surgery-support devices, but by capturing the anatomic features in surgical field, or in other words, by touching the pedicle by some method. These efforts can be broadly divided into 2 methods: one is to confirm the outer wall of the pedicle from the spinal canal side, and the other is to confirm the inner wall of the pedicle from the inside the bone. There have been reports on the method of palpating the outer wall of the pedicle from the spinal canal side under direct vision by resecting part of the lamina or facet, followed by placement of a CPS while reviewing the position of the pedicle.^[13,14]^ However, this method may possibly cause bleeding from the epidural venous plexus due to palpation or spinal cord and nerve root injuries in case spinal canal stenosis is present at the site. On the other hand, the method suggested by Abumi et al includes drilling the cervical lateral mass in a funnel shape, palpation of the inlet of the pedicle from the inside of the bone using a tool such as a probe, and placing a CPS while reviewing the position of the pedicle.^[15,16]^ There have been reports on the application of the method at the thoracic spine level.^[17,18]^ Another known method at the thoracic spine and lumbar levels is the ball-tip method, in which a PS is placed while reviewing the palpable feel of the spinal canal wall and the inner wall of the pedicle with the tip of the progressing probe without drilling the bone.^[19]^ The method of direct visualization of the outer wall and inner margin of the pedicle while reviewing it with a C-arm, as well as that of placing a PS while reviewing the position of the pedicle through palpation with a probe or other tools, are superior in safety, and their accuracy is guaranteed to a certain extent. In order to further improve safety with reference to the idea of palpation of the pedicle, we devised a method of directly exposing the LE points, which are the lateral edge of the cortical bone of the spinal canal, while reviewing the position under lateral fluoroscopy, and confirming the site reliably. We then applied the method to CPS placement surgery, which is introduced in the present case report. A U-shaped wire can be prepared inexpensively and easily in a short period of time, and it is a useful tool that enables safe placement of a CPS. Considering that LE points are an important anatomical feature for PS placement, the bone is first drilled with a high-speed drill to expose the site. Here, the key is that the use of a U-shaped wire confirms that the exposed site corresponds to the LE points that can be reviewed on preoperative CT images. Furthermore, it can be confirmed under lateral fluoroscopy that the ends of the U-shaped wire correspond to the LE points, and thus, the method is equipped with multiple “check” mechanisms that enhance safety. As the bone at the placement site is shaved, the contact area between the screw surface and the bone becomes smaller, raising a considerable concern in terms of fixedness. However, we believe that fixedness is ensured by placement of a screw while anchoring it in the thick cortical bone inside the pedicle. Although the drilling site in the cephalad side of the lamina serves as the bone graft bed, new bone formation around the screw can be expected by firm placement of the grafted bone at the drilling site present around the screw. The use of the U-shaped wire is considered difficult in the following cases: prominent osteosclerosis or deformity around the pedicle, destroyed lateral mass or pedicle from the lamina due to trauma, and vertebral artery running through the transverse foramen that extends into the lateral mass. A limitation of this study is that it reports a single case, and further studies with a larger number of cases are needed to confirm the superiority of this technique.

## 4. Conclusion

Surgery for cervical myelopathy with kyphosis, including the placement of CPSs at C3 without the occurrence of complications, can be easily performed by using a U-shaped wire capable of identifying LE points under direct vision and reliably confirming the site with C-arm lateral fluoroscopy.

## Acknowledgements

We would like to thank Editage (www.editage.com) for English language editing.

## Author contributions

**Data curation:** Tomoaki Kanai, Naomu Sawada.

**Formal analysis:** Chikara Ushiku, Shoshi Akiyama.

**Investigation:** Chikara Ushiku.

**Writing – original draft:** Chikara Ushiku.

**Writing – review & editing:** Chikara Ushiku, Mitsuru Saito.
